# Is Alzheimer's disease an individual-centered disease? Hypotheses from the atomic levels up to mathematical models for biological systems

**DOI:** 10.3389/fneur.2024.1352261

**Published:** 2024-02-28

**Authors:** Maurizio Giorelli, Donatella Accavone, Alfredo De Liso

**Affiliations:** Operative Unit of Neurology, Azienda Sanitaria Locale Barletta-Andria-Trani (ASL BT), Barletta, Italy

**Keywords:** Alzheimer's disease, neuropathological biomarkers, amyloid-beta, risk factors, Boolean (control) network, co-pathologies

## Introduction

Alzheimer's disease (AD) is a dementing affection that is neuropathologically characterized by the deposition of amyloid-beta (Aβ) plaques and neurofibrillary tangles (NFTs), which in turn cause neurodegeneration and the onset of clinical symptoms (1). Aβ derives from the sequential processing of Amyloid Precursor Protein (APP) by β and ⋎ secretase. APP peptides, such as Aβ1–42 or Aβ1–40 may form oligomers, small aggregates, or fibrils and damage neurons by inducing oxidative stress, suppressing the function of membrane channels, or affecting transport/sorting mechanisms. It is believed that Aβ1–42 causes the hyperphosphorylation of tau, which in turn leads to fibrils aggregation and neurotoxicity (2). The past few decades saw an overgrowth of studies aiming to elucidate AD etiology and promoting development of new Disease Modifying Drugs. Besides these tremendous efforts, the full understanding of AD etiology and pathogenic cascade is still hiding. Several studies have demonstrated that other misfolded proteins do exist in the brain of AD patients as a rule, not an exception (3). It has been hypothesized that pathologic misfolded proteins may promote synergistic pathology and reciprocal misfolding and aggregation (4). The mutual influence of pathogenic mechanisms triggered by different abnormal proteins can therefore shape AD leading to a plethora of neuropathological variants. In this paper we postulate that AD variants arise from a non-linear regulation of pathogenic mechanisms as those observed for complex biological networks (5).

## The multiplicity of pathogenic mechanisms in AD

Multiplicity of pathogenic paths associated with AD may well explain the neuropathological splitting of AD variants. AD may be triggered by mutation within several genes such as PSEN (presenilin)1, PSEN (presenilin) 2, APP (Amyloid Precursor Protein) (2, 6). Genome-wide association studies (GWAS) and Whole Exome Sequencing (WES) have further identified several other AD-associated rare variants, particularly in Familial-AD (6–8). Neuro-inflammation plays a central role in the pathogenesis of AD (4). Initial deposition of Aß may induce persistent activation of microglia. Release of proinflammatory cytokines such as IL-*1ß* and activation of Toll like receptors from activated microglia impairs branching of dendritic spines and disrupts microglial-induced scavenging of Aß (4). The Gut Microbioma (GM) is the population of bacteria, viruses, fungi and protozoa residing in the human gut. It is unique to each individual and is involved in the regulation of the immune axis and in responses to stress. Specific GM pedigrees have been found to be associated with neurodegenerative diseases such as Parkinson's disease (9) and AD (10). Other pathogenic mechanisms involved in AD are: tau hyper-phosphorylation; decreased affinity to microtubules; formation of NFTs; formation of reactive oxygen species; or infections due to HSV-1, spirochetes, or chlamydia (3).

All these pathogenic mechanisms, either genetic or epigenetic in nature, may induce or suppress transcription of specific genes, enhance unpredictable proteomic interactions, activate o turn off target enzymes, and ultimately lead to functional consequences on neuronal metabolism that can amplify downstream in a “cascade” effect (6, 11). The network of enzymatic paths leading to dysfunction of neuronal plasticity and cell death does not simultaneously activate as a whole. Rather, specific branches of the pathogenic cascade switch on or off specifically and finely as a result of a continuous range of activation of each specific enzymatic node. The interaction of multiple pathways can therefore modulate neurodegeneration and induce complex neuropathology.

## Pleomorphism of AD: typical and atypical AD

The classic presentation of AD (i.e., typical AD dementia) is a slowly progressive amnestic disorder associated with the degeneration of medial temporal lobes, which then progresses to involve distant cortical structures, thus leading to multi-domain dementia. At variance with this classic AD variant, non-amnestic phenotypes of AD have been identified so far whose brain degeneration prevails mostly within the frontal, parietal or occipital lobes being clinical syndromes characterized by dominant difficulties in visual, language, executive, behavioral, and motor domains (1). The typical and atypical forms of AD differ in their patterns of neurodegeneration but share similar pathogenic mechanisms, ultimately converging on the final nucleation and aggregation of Aβ. The molecular reasons for these differences may be subtended by a greater tau burden in younger patients affected by atypical forms of AD (12). The variability in tau burden and its distribution explains the failure in different brain networks (13), but ultimate reasons for such a clinical landscape remain obscure. Several mechanisms have been invoked to explain such a pleomorphism and span from the co-presence of additional abnormal proteins (co-pathologies) within the AD brain up to the existence of distinct structures of Amyloid fibrils.

## Multiple co-pathologies and risk factors

Other pathological conditions coexist with Aβ in 98% of early-onset AD (EOAD) and in 100% of late-onset AD (LOAD) (3). Co-pathologies include Cerebral Amyloid Angiopathy (CAA), Lewy Body disease (alpha-synucleinopathies), Limbic-predominant age-related TPD-43 (LATE), Hippocampal Sclerosis (HIS), Argyrophilic Grain Disease (AGD), vascular disease, Aging-related tau astrogliopathy (ARTAG), and Argyrophilic Thorny Astrocytes in clusters (ATAC) (3, 14).

Synuclein-enriched Lewy body disease (LBD) is the most common co-pathology in AD and may contribute to clinical onset and status (3, 15, 16). Reciprocal interactions of Aβ with either α- synuclein (α- syn), TPD-43, and tau have been suggested by some evidence. Interestingly, the presence of co-pathologies increases in the late stage of AD, thus suggesting that distinct pathologic mechanisms may influence each other. The most claimed hypothesis is that misfolded proteins can cross-seed the aggregation of additional proteins (cross-seeding) by acting as template for their seeding and by enhancing the conformational distortion of oligomers and fibrils (4). In addition, distinct misfolded proteins may co-aggregate and further promote synaptic dysfunction and neurodegeneration (4). As a third mechanism, interaction of monomers from distinct proteins may induce post-translational modifications such as tau phosphorylation and the collapse of neuronal cytoskeleton (4). Downstream activation of metabolic pathways may finally lead to cellular apoptosis.

Cerebrovascular disease frequently coexists with Aβ. Damage to the small cerebral vessels (arteriolosclerosis) favors the deposition of extracellular Aβ which, conversely, worsens vessel sclerosis by depositing itself in the arterioles (17). Cerebrovascular disease is characterized by enormous genetic pleomorphism and sensitivity to risk factors. For example, hypertension, which can promote damage to small cerebral vessels, is modulated by age, genes, drugs and variable factors linked to lifestyle habits. Modifiable risk factors can vary over the course of an individual's life. For example, a patient may quit smoking or start drinking alcohol heavily. These variations affect the progression of vascular damage and the deposition of Aβ. Little is known about the possible change in risk in patients who move away from their birthplace possibly acquiring new eating or voluptuary habits or being exposed to environmental pollution that can increase cerebrovascular damage. Therefore, the rate of accumulation of vascular or amyloid-related brain damage may change over time during the life span (15).

## Amyloid fibrils

Cryo-Electron Microscopy (CEM) of brain-derived amyloids found at least four different types of Aβ fibrils arising from the sequential cleavage of Amyloid APP by β- and ⋎-secretases (18). The Tau protein exists in six isoforms of which three contain all four microtubule-binding repeats, while the other three isoforms have only three repeats, lacking the R2 repeat (18). Many neurodegenerative diseases result from the abnormal assembly of tau and are therefore called tauopathies. Differential composition of filaments isoforms results in distinct neurodegenerative diseases. In fibrils from AD all six isoforms exist, whereas filaments include only 4R isoforms in Corticobasal Degeneration and Progressive Supranuclear Palsy (18). Alpha-synuclein (α-sin), on the other hand, exists in three different filaments folds. Combination of these filaments results critical for determination of either the pattern of neurodegeneration and clinical phenotype (18–20). It has been further demonstrated that environmental factors such as temperature, salt concentration, pH, or agitation may induce higher level conformational changes in the nucleated amyloid seed thus leading to variation in fibrils composition (21). Possibly, environmental factors throughout the individual's life may shape fibrils composition in the aggregated Aβ, α-syn and TPD-43. The complexity and the number of co-pathologies in AD, and the plethora of conformational isoforms that may contribute to form fibrils of these misfolded proteins, may well explain the shaping of the neuropathological variants in AD subtypes.

## Resistance to anti aβ- monoclonal antibodies

Lecanemab and aducanumab are monoclonal antibodies directed against oligomeric Aβ-peptides and capable of reducing amyloid deposits in the brain in early onset AD. General results from trials exploring the effects of lecanemab showed small significance and raised concerns on its clinical relevance in clinical practice (22, 23). By contrast, aducanumab was approved on the basis of its ability to reduce Aβ deposits in the brain despite an interim analysis in 2019 that demonstrated its futility (24).

The eligibility of anti-Aβ monoclonal antibodies is based on the idea that Aβ-associated neuropathology “explains” the symptoms. Both brain imaging and the biological fluids–associated markers of AD have been used to assess the patient's eligibility to DMDs such as Aβ scavenging therapeutics (22–24). Different factors may underlie the partial non-responsiveness of AD to anti-amyloidotic therapies. Both co-pathologies and the existence of conformational variants of abnormal proteins can modulate the deposition of fibrillary Aβ, the pattern of neurodegeneration, and the clinical phenotype of the resulting dementia (4, 15). The coexistence of mixed pathology may dilute the protective effect of anti-Aβ scavenging therapies. Brain Positron Emission Tomography (PET) for Aβ recognizes the extra-neuronal fragment of Aβ, the Aβ deposited in the vessels, or both (25). This might be a relevant bias when searching for the imaging biomarkers of AD and when testing eligibility to therapeutics that are specific for the neuronal deposition of Aβ. The presence of intravascular amyloid may lead to the overestimation of results from brain PET, thus categorizing variants that are mixed by a neuropathological point of view as “pure” Aβ-related AD forms (14). This “dilution effect” may reduce the effect of anti-Aβ monoclonal antibodies. A further criticism toward the adoption of Aβ as AD biomarker is the fact that, among elderly patients with cerebral amyloid, some patients experience the accumulation of further Aβ and develop cognitive impairment, whereas others remain unaffected (26, 27). This progression profile remains elusive at beginning.

## Network models of biological systems

**Figure 1:**
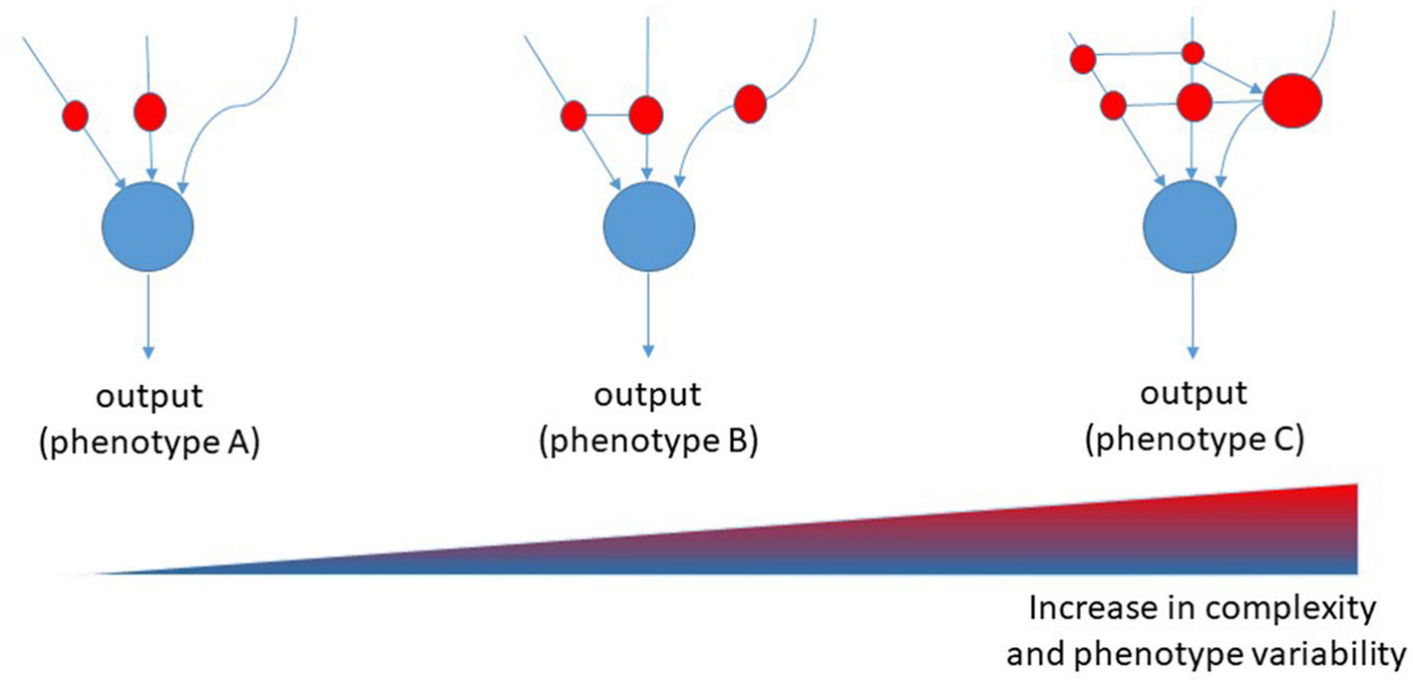
Proposed Boolean network model for diseases. Red cycles represent nodes of interaction among causative, predisposing, and risk factors. The size of the red circles represents the strength of involvement of that node, whereas connectors represent the transversal regulation of nodes [modified from (28)].

**Figure 2:**
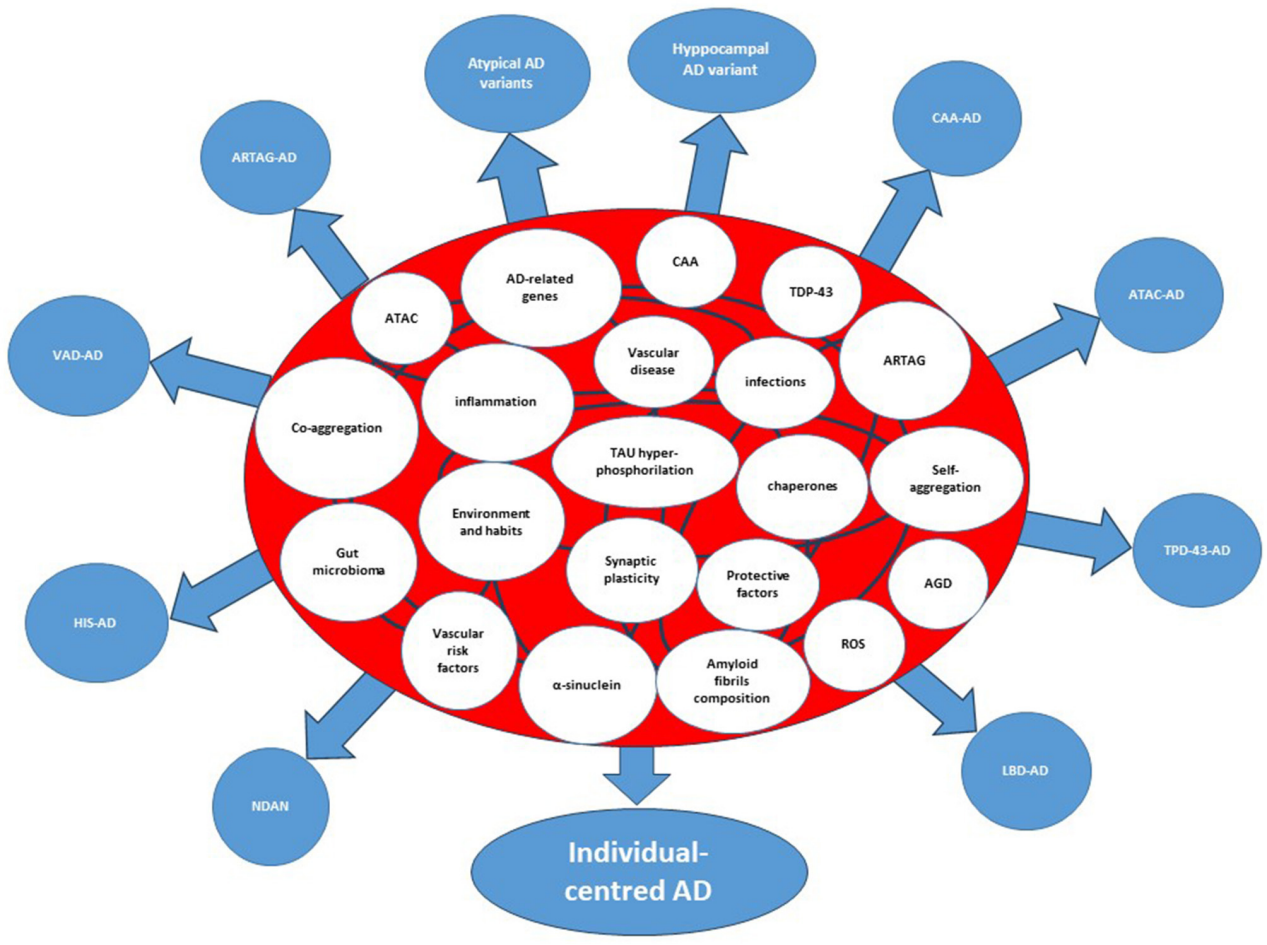
Interactome and splitting of the clinicopathological forms of Alzheimer's disease. The etiologic, predisposing, and risk factors within the circles represent the nodes of the system. The curved lines connecting the circles represent interactions among nodes. AD, Alzheimer's disease; AGD, Argyrophilic Grain Disease; ARTAG, Aging-related TAU astrogliopathy; ATAC, Argyrophilic Thorny Astrocytes in Clusters; CAA, Cerebral Amyloid Angiopathy; CDR-SoB, Clinical Dementia Rating-Sum of Boxes; HIS, Hippocampal Sclerosis; LATE, Limbic-Predominant Age-Related TDP-43 encephalopathy; LBD, Lewy Body Disorders; VAD, Vascular Disease; NDAN, Non-Demented with Alzheimer's Neuropathology; Hyppocampal AD variant=amnestic AD; atypical AD variants, Posterior Cerebral Atrophy, lvPPA (logopenic variant Primary Progressive Aphasia, Cortico-Basal-Syndrome AD, dyxesecutive AD.

It is tempting to suggest that the complexity of AD follows the models of “nonlinear regulation,” which have been demonstrated for biological networks (5). These models have been set on the basis of Boolean networks models, which are interacting nets characterized by a cascade of events that regulate each other with variable strength and connections. Each node in a Boolean network can only be in one of two states, namely, on or off. However, its state of activity is critically influenced by all inputs acting on the node. Inputs vary in terms of strength and numbers and may change over time. Therefore, the final output of the network (the result) can vary greatly. When translated to a biological network, the result is that the number of biological variants depend on the number and strength of nodes, and from their interactions. Thus, the many are the nodes and the interactions, the many are the resulting variants, the more is the complexity of that biological system (Figure 1). In the case of AD, either causative, predisposing, facilitating, protecting, or regulating factors represent nodes within a patient's specific network. Indeed, each patient has his own fingerprint of AD-associated factors, either genetic or acquired, which may interact with variable strength and duration during the life course. The outcome, i.e., that neuropathological variant of AD, will be the result of the selected final path arising from the patient's interactome. Considering the biological evidence collected to date and given the described Boolean model, AD should be considered an individual-centered disease rather than an Aβ-centered disease with multiple clinical faces (Figure 2). Patient's AD might arise from the one's specific regulation of personal genetic and environmental risk factors around nodes that interact in a continuous but variable fashion. Thus, the concept of “one patient-one AD variant” might better describe the enormous pleomorphism of AD rather than the “one-size-fits-all” concept. Precision medicine should be used in assessing diagnoses such as AD that involve many causative genes, proteins, and risk factors as biomarkers (29). Collection of lab results, medical questionnaires and formal clinical evaluation is critical to unravel the one's specific contributors to cognitive decline. An individually-tailored program aimed to counteract specific risk factors demonstrated to be effective in reversing subjective or mild cognitive impairment in a precision medicine perspective (30). Such an approach would allow the exact characterization of each single patient's burden of pathogenic factors thus leading to the adoption of an individualized therapeutic strategy.

## Author contributions

MG: Conceptualization, Formal analysis, Investigation, Writing – original draft. DA: Validation, Writing – review & editing. AD: Validation, Writing – review & editing.
